# Bacterial Meningitis in Infants Under 90 Days of Age: A Retrospective Single-Center Study

**DOI:** 10.3390/children11121411

**Published:** 2024-11-22

**Authors:** Martina Buttera, Sofia Mazzotti, Tommaso Zini, Lucia Corso, Valeria Dallai, Francesca Miselli, Luca Bedetti, Katia Rossi, Eugenio Spaggiari, Lorenzo Iughetti, Licia Lugli, Alberto Berardi

**Affiliations:** 1 School of Pediatrics Residency, University of Modena and Reggio Emilia, 41224 Modena, Italy; 297187@studenti.unimore.it (M.B.); 196397@studenti.unimore.it (L.C.); lorenzo.iughetti@unimore.it (L.I.); 2 Pediatric Unit, Arcispedale Santa Maria Nuova, University of Modena and Reggio Emilia, 41224 Modena, Italy; tommaso.zini@unimore.it; 3 Degree Program in Medicine and Surgery, University of Modena and Reggio Emilia, 41224 Modena, Italy; 213636@studenti.unimore.it; 4 Neonatal Intensive Care Unit, University Hospital of Modena, 41224 Modena, Italy; 79638@studenti.unimore.it (F.M.); bedetti.luca@aou.mo.it (L.B.); rossi.katia@aou.mo.it (K.R.); spaggiari.eugenio@aou.mo.it (E.S.); lugli.licia@aou.mo.it (L.L.); alberto.berardi@unimore.it (A.B.); 5 Pediatric Unit, University Hospital of Modena, 41124 Modena, Italy

**Keywords:** meningitis, bacterial meningitis, neonatal infection

## Abstract

Background: Bacterial meningitis (BM) in infants is a serious condition that can lead to significant complications. Lumbar puncture (LP) is essential to provide diagnoses, however false negatives may result if LP is performed after the starting of antibiotic therapy. Methods: We conducted a retrospective analysis of infants of any gestational age with BM within their first 90 days of life and admitted to the Neonatal Intensive Care Unit of Modena Policlinico between 1 January 2011, and 31 December 2023. Results: A total of 44 episodes of meningitis were confirmed in 40 infants, diagnosed by positive cerebrospinal fluid cultures (*n* = 37), polymerase chain reaction testing (*n* = 4), or both methods (*n* = 3). Three out of forty infants (8%) experienced a relapse of meningitis. Most episodes (31/44, 70%) occurred in preterm infants. The incidence of early-onset meningitis was lower than that of late-onset (0.18 vs. 0.94 cases per 1000 births, respectively), with Gram-positive accounting for most cases (27/44, 61%). LP was performed prior to antibiotic administration in most episodes (30/44, 68%). Two preterm infants (5%) died from meningitis-related complications. Forty-two episodes occurred among thirty-eight surviving infants; brain lesions were detected through brain ultrasound or MRI in nine out of forty-two episodes (21%). Conclusions: Preterm infants have higher rates of BM, brain lesions or case fatalities. Early diagnosis and prompt antibiotic treatment are critical to improve outcomes.

## 1. Introduction

Meningitis is an inflammatory disease caused by microbial infection [1]. Neonates and infants up to 90 days old are particularly vulnerable to sepsis and meningitis, with the highest incidence occurring within the first month of life [2]. Neonatal bacterial meningitis (BM) affects 0.1–0.4 per 1000 live births, but rates are higher in chronically hospitalized and preterm infants, especially those with very low birth weight (VLBW), reaching up to two per thousand live births [3]. The true incidence may be underestimated, as 30–50% of neonates admitted to intensive care units for sepsis do not undergo lumbar puncture [4,5]. BM typically originates from primary bacteremia, which spreads to the central nervous system; as a result, meningitis co-occurs with sepsis in 25–60% of cases [6]. Key risk factors for BM include the immaturity of the neonatal immune system and low levels of maternal antibodies [7]. Neonatal meningitis, like sepsis, is classified into early-onset meningitis (EOM, occurring within the first 72 h of life), and late-onset meningitis (LOM, occurring from 72 h onwards) [8,9,10]. The epidemiology and causes of bacterial meningitis have evolved over the past 20 years due to the availability of effective vaccines targeting major pathogens. Intrapartum antibiotic prophylaxis (IAP) has also reduced the incidence of early-onset sepsis caused by Group B Streptococcus (GBS). Despite these advances, GBS remains a major neonatal pathogen, responsible for over 40% of EOM cases [4,11,12], with an incidence ranging from 0.14 to 0.41 per 1000 live births [8,13]. *Escherichia coli* is the second most common pathogen, accounting for approximately 30% of EOM cases, followed by *Listeria monocytogenes* [4,14,15].

Diagnosing meningitis in neonates is challenging due to its nonspecific initial symptoms, which overlap with those of sepsis. Symptoms may include hypothermia or fever, delayed capillary refill, vomiting or poor feeding, respiratory distress or apnea, jaundice, diarrhea, lethargy or irritability, bulging fontanelle, seizures, and neck stiffness [16]. Hematologic tests, such as white blood cell count (WBC), C-reactive protein (CRP), and procalcitonin (PCT) are low predictive and without performing a lumbar puncture (LP) they do not allow the ruling out of BM. Furthermore, LP is essential to identify the pathogen (via culture or molecular methods like polymerase chain reaction, P.C.R.) and determining antibiotic susceptibility for targeted treatments [17].

When BM is suspected, treatment should be promptly and aggressively initiated. Empirical antibiotic therapy typically includes a combination of a beta-lactam (such as ampicillin) with an aminoglycoside or cefotaxime for infants under 30 days old and ampicillin with cefotaxime (or ceftriaxone or vancomycin) for those aged 30 to 90 days [18]. Antibiotics should be immediately administered i.v. after LP to ensure rapid cerebrospinal fluid (CSF) sterilization [19,20,21]. Delays in treatment increase mortality and morbidity, leading to complications such as hypotension, cerebral infarctions, seizures, and increased intracranial pressure [22]. Approximately 10% of neonatal BM cases are fatal or result in neurological complications, including infarctions, hemorrhages, intracerebral thrombosis, and brain abscesses. Up to 50% of cases lead to long-term sequelae, such as intellectual disability, hydrocephalus, epilepsy, cerebral palsy, cortical deafness, or cortical blindness, with varying severity [23,24,25,26,27].

Magnetic Resonance Imaging (MRI) is recommended for neonates with persistent positive CSF cultures despite antibiotic treatment, new or ongoing seizures, or abnormal neurological findings. Common MRI findings include leptomeningeal enhancement (57% of cases), cerebral infarctions (43%), subdural empyema (52%), encephalitis (26%), hydrocephalus (20%), and abscesses (11%) [28].

The aim of this study is to analyze a cohort of infants diagnosed with BM and admitted to our hospital, with a focus on epidemiology, clinical presentations, antibiotic treatments, and short-term outcomes.

## 2. Materials and Methods

This is an observational, retrospective, non-funded, monocentric study conducted on infants of any gestational age affected by BM under 90 days of life. Infants were admitted to the NICU of Modena Policlinico Hospital (1 January 2011–31 December 2023). This is a high-volume level-three facility, with inborn neonates accounting for most admissions. The NICU contains 20 cots, receives approximately 450 admissions per year and the medical staff consists of 12 physicians. The population of infants admitted to the NICU mainly includes premature newborns, with special attention to post-surgery patients (coming from other wards) or infants being transferred from other facilities.

### 2.1. Inclusion and Exclusion Criteria

The inclusion criteria were (i) inborn infants (ii) with at least one CSF culture or P.C.R. positive for a bacterial pathogen. Cases were identified by using the database provided by the Microbiology and Virology laboratory.

### 2.2. Data Collection

The following data regarding maternal and obstetric information were collected: delivery mode, group B streptococcus (GBS) screening and intrapartum antibiotic prophylaxis (IAP), date of birth, neonatal gender, gestational age, birth weight, 5 min Apgar score, clinical symptoms at the onset on infection, laboratory tests, brain imaging (ultrasound or cerebral MRI), therapies, antimicrobial susceptibilities of pathogens, need of catecholamines support or mechanical ventilation.

All data were entered in an anonymous Excel spreadsheet for Microsoft 365 (version 2006) with controlled access, assigning a progressive numerical code to each newborn. For each patient we fulfilled a comprehensive set of data encompassing demographic and clinical characteristics at birth and during hospital stay.

### 2.3. Definitions

- Full-term and preterm neonates: neonates with gestational age ≥ 37 weeks and <37 weeks, respectively.
- Extremely preterm neonates: neonates with gestational age < 28 weeks.
- Extremely low birth weight (ELBW): birth weight < 1000 g.
- Early- (EOM) and late-onset meningitis (LOM): presence of clinical signs of meningitis and isolation of the pathogen from CSF within or after 72 h, respectively.
- Culture-proven meningitis: positive CSF culture or P.C.R. test for a pathogen obtained from CSF, in infants with clinical symptoms of meningitis and specific antibiotic treatment.
- Polymicrobial infection: presence of more than one pathogen in CSF.
- Relapse of the infection: a pathogen cultured at least 7 days after the initial infection.
- Antimicrobial susceptibility: susceptibility was determined according to laboratory results. For cases where diagnosis was made by P.C.R., susceptibility for certain pathogens (e.g., *Listeria monocytogenes* or GBS) was established a priori, based on the antimicrobial susceptibility of isolates previously tested in the NICU.
- Antimicrobials: empirical antibiotics were administered within the first 72 h after obtaining blood and/or CSF samples. When possible, targeted single-agent therapy was given after empirical therapy based on pathogen susceptibility results.
- Meningitis-related brain lesions: brain lesions (white matter damage, hydrocephalus, ventricular enlargement, and intraventricular hemorrhage of grade ≥ 2 according to Papile classification) were identified shortly after the meningitis episode through MRI or brain ultrasound.
- Meningitis due to coagulase-negative staphylococci (CoNS): in line with the more recent literature, CoNS yielded from CSF were included if infants received targeted antimicrobials, showed clinical signs of sepsis/meningitis, and the medical team diagnosed BM [29,30].

### 2.4. Laboratory Analyses

C-reactive protein (CRP) levels were considered normal when below 1 mg/dL [31]. The following CSF parameters were considered “normal” (below the 95th percentile) for preterm infants: white blood cell count ≤16 cells/μL, protein ≤203 mg/dL, and glucose ≥33 mg/dL. For full-term infants, normal values were defined as a white blood cell count ≤26 cells/μL, protein ≤137 mg/dL, and glucose ≥36 mg/dL [32].

### 2.5. Statistical Analysis

Statistical analysis was performed using “Medcalc” for Windows (version 9.3.0.0, 2007). Continuous variables, such as weight and gestational age, were reported as mean, median, standard deviation, and interquartile range (IQR); categorical variables were reported as absolute numbers and percentages.

Continuous variables were analyzed using a non-parametric test (Mann–Whitney U test). The threshold for statistical significance was set at a *p*-value of <0.05.

### 2.6. Ethical Considerations

This study was conducted in accordance with the Declaration of Helsinki, Edinburgh Revision (2000). The study was approved by the Area Vasta Nord-Emilia Romagna Ethics Committee (protocol number 0028835/24). Given the impossibility of retrieving retrospectively the consent of all the infants included in this study, the research ethics committee waived the need for consent.

## 3. Results

### 3.1. Study Population

**Figure 1:**
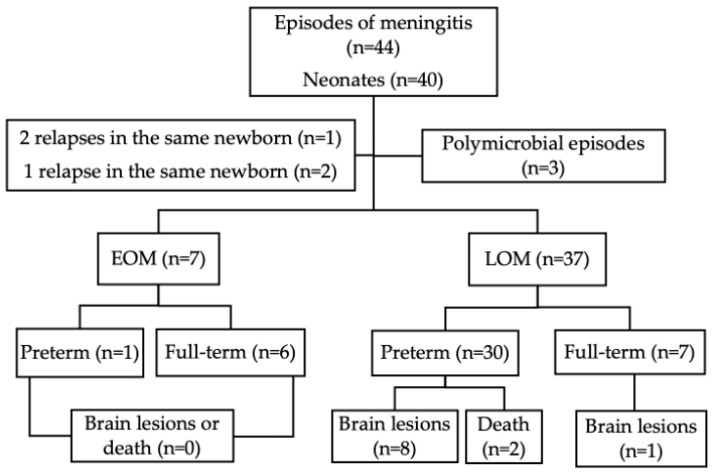
Main characteristics of the study population. EOM: early-onset meningitis; LOM: late-onset meningitis.

Over the 13-year study period, a total of 39,150 neonates were delivered. Of these, 40 infants were diagnosed with BM. Among them, 21 (53%) were males and 19 (47%) were females; 13 (32%) were full-term and 27 (68%) were preterms, of which 11 (28%) were ELBW. Three neonates experienced meningitis relapses: two had a single relapse, and one had two relapses, resulting in a total of 44 confirmed episodes of BM. Among these, seven episodes (16%) were classified as EOM and 37 (84%) as LOM. All infants who relapsed had a ventricular shunt. In three of the forty-four episodes (7%), the infection was polymicrobial. Figure 1 provides an overview of the study population’s main characteristics and infant outcomes.

The overall incidence of meningitis was 1.12 cases per 1000 births, with rates of 0.18 and 0.94 cases per 1000 births for EOM and LOM, respectively. The median gestational age for EOM cases was 39 weeks (IQR: 37.25–39.75), while for LOM cases was 28 weeks (IQR: 26–32). Of the 37 LOM cases, 32 (87%) occurred during their hospital stay, while 5 (13%) developed after discharge and were therefore considered community-acquired infections. Blood cultures were performed simultaneously with the initial LP in 39 of 44 episodes (88%) and were positive in 28 out of 39 episodes (72%). In 23 of the 28 positive episodes (82%), the same pathogen was identified in both blood and CSF cultures, whereas in the remaining five episodes (18%), different pathogens were detected. Table 1 details the pathogens found in blood and CSF cultures for each episode, along with information on relapses and clinical outcomes, including brain lesions or death.

### 3.2. Pathogens Responsible of EOM and LOM

**Figure 2:**
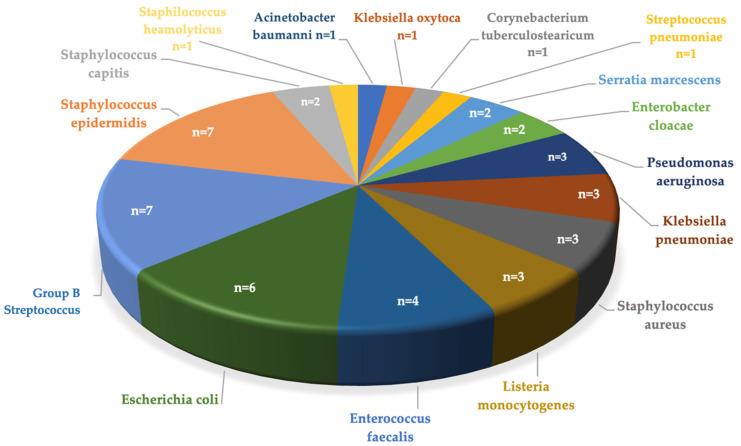
Pathogens isolated from 40 neonates with 44 episodes of bacterial meningitis, diagnosed through CSF culture or P.C.R.

A total of 47 pathogens were identified through CSF culture or P.C.R., including three cases of polymicrobial infection (see Table 1 and Figure 2 for details). Gram-positive bacteria accounted for the majority of meningitis episodes (27/44, 62%), while Gram-negative bacteria were responsible for 34% of cases (15/44). The remaining 4% (2/44) involved both Gram-positive and Gram-negative pathogens.

For EOM, the most frequently identified pathogens were GBS (*n* = 2) and *Listeria monocytogenes* (*n* = 2). In contrast, LOM wase most often caused by *Staphylococcus epidermidis* (*n* = 7) and GBS (*n* = 5) (see Table 1).

### 3.3. Clinical Findings

The most commonly reported initial symptoms in preterm infants were apnea (*n* = 15), irritability or poor responsiveness (*n* = 12), heart rate changes (*n* = 9), and desaturation (*n* = 7). In contrast, term infants more frequently presented with irritability or poor responsiveness (*n* = 6), respiratory symptoms (*n* = 4), and elevated temperature (*n* = 3) at disease onset.

Mechanical ventilation was initiated at symptom onset in 15 (35%) out of 43 episodes of BM (one episode was excluded because the infant was already on mechanical ventilation), with a median duration of 5 days (IQR: 2–14). Catecholamine support was required in 17 out of 44 episodes (39%).

Brain ultrasound study was performed for all 40 infants after each BM episode; no brain lesions were observed after the episode in 29 out of 42 episodes (69%, excluding deceased infants). In four of the forty-two episodes (10%), pre-existing brain lesions were detected, which remained unchanged after BM. Thus, new brain lesions developed following BM in nine of forty-two episodes (21%) (see Table 1 and Figure 1 for details). The most common findings included white matter hyperechogenicity or hyperintensity and ventricular enlargement, present in all neonates with brain lesions. Brain MRI was conducted in only 31 of the 38 surviving infants (82%, two infants died).

At the time of BM diagnosis, five out of forty neonates (13%) already had a ventricular-peritoneal shunt in place, while two infants (5%) required shunt placement after the BM episode.

### 3.4. Analyses of CRP Levels, WBC Values and CSF Parameters

C-reactive protein (CRP) levels at the clinical onset of BM were measured in 34 out of 44 episodes (77%). Of these, CRP was positive (≥1 mg/dL) in 27 episodes (79%). WBC counts at onset were available in 35 out of 44 episodes (80%), with WBC counts exceeding 5000/mm^3^ in 23 episodes (52%).

In 14 out of 44 episodes (32%), LP was performed after the initiation of empirical antibiotic therapy, with a median delay of 24 h (range: 1–48 h, IQR: 2.25–24 h).

CSF parameters at symptom onset were available for 33 out of 44 episodes (75%); in the remaining 11 episodes (25%), traumatic lumbar puncture prevented data analysis. CSF white blood cell counts were within the normal range in 13 out of 44 episodes (30%), with a median WBC count of 33 cells/mm^3^ (range: 1–50,830; IQR: 8.5–510).

CSF protein levels at symptom onset were available in 29 out of 44 episodes (66%), with normal protein levels observed in 15 episodes (34%). The overall median CSF protein level was 200 mg/dL (range: 70–1200 mg/dL; IQR: 101–300 mg/dL).

CSF glucose levels at onset were available in 30 out of 44 episodes (68%), with normal glucose levels documented in 12 episodes (27%). The overall median CSF glucose level was 37 mg/dL (range: 20–97 mg/dL; IQR: 20–59 mg/dL). Additional information on blood and CSF parameters is provided in Table 2.

### 3.5. Empirical and Target Therapy

Antimicrobial susceptibility testing was conducted for each positive culture, allowing for the administration of narrow-spectrum antibiotics as soon as susceptibility results became available. For infants with positive CSF cultures and those with GBS or *Listeria monocytogenes* detected by P.C.R., antimicrobial susceptibility was assessed.

In 34 out of 40 episodes (85%), the pathogen was susceptible to at least one of the initial empirical antibiotics. This analysis included episodes diagnosed through a positive CSF culture (*n* = 37) or P.C.R. (*n* = 3). However, effective empirical antibiotics (with an ideally adequate blood-brain barrier penetration) were administered in only 36 (82%) out of 44 episodes. As a result, empirical therapy was considered suboptimal in eight episodes (18%).

The most frequently administered empirical regimen was a combination of an aminoglycoside and a beta-lactam, used in 28 out of 44 episodes (64%). For targeted therapy, the most commonly used antibiotics for Gram-positive infections included a combination of a beta-lactam (penicillin or oxacillin) and an aminoglycoside (gentamicin or amikacin) in twenty-one cases, a glycopeptide and an aminoglycoside in four cases, a switch from beta-lactam and aminoglycoside to glycopeptide and/or cephalosporin in four cases, and various other combinations in fifteen cases. For Gram-negative infections (15 episodes), a cephalosporin was added in nine cases, a carbapenem in five cases, and empirical therapy was maintained in two cases. In two episodes, CSF was polymicrobial with both Gram-negative and Gram-positive bacteria.

The median duration of antibiotic therapy was 14 days (range: 2–35 days, IQR: 10–20.5 days). Four cases were excluded from this calculation: two infants who died early in treatment and two who had already been on prolonged antibiotic therapy. Preterm infants were not more likely to receive extended antibiotic courses (median duration in preterm infants 14 days, range 3–32 days, IQR: 10.5–19.5 days, vs. full-term infants 14 days, range 2–35 days, IQR: 7–20.5 days).

## 4. Discussion

In this study, the overall incidence of both EOM and LOM was 1.12 per 1000 live births, that is higher than incidences (approximately 0.3 cases per 1000 live births) reported in developed countries such as the Netherlands and Sweden [18,33]. In contrast, a wide study conducted in Turkey (encompassing infants of all gestational ages and using NICU admissions as the denominator) reported a higher incidence (2.51 per 1000 cases) [29]. In addition, a recent multicenter Chinese study focused on preterm neonates under 32 weeks of gestation found an incidence of 3.8 per 1000 live births [30]. Overall, the comparison with other studies is difficult due to the heterogeneity of the populations and selection criteria.

In our cohort, 84% of BM episodes were LOM. This predominance is not unexpected, as our center follows widespread protocols for IAP to prevent GBS. Consistent with previous literature, BM incidence is higher in preterm neonates, who accounted for 68% of all episodes. Most infants had a blood culture performed at the onset of symptoms, which were positive in 72% of episodes, with a high concordance rate (82% of cases) between blood and CSF culture. Our findings align with those of a multicenter study that included 150 NICUs and analyzed data from 9111 neonates with ≥34 weeks’ gestation; this study reported that 62% of neonates with BM also had positive blood cultures [34].

Case fatality rates after BM may reach 50% of cases in earlier studies and mostly mortality occurs in preterm infants [13]. In the current study, only two neonates (5%), both extremely premature died; this case fatality rate is comparable to the 13% recently reported by Baud who also report an almost double risk (25%) in preterm neonates [6].

Long-term outcomes of BM may vary. Baud reports poor outcomes in 20–50% of meningitis survivors when a long-term follow-up is performed [6]. A prospective study including >1500 neonates with BM, and examining children until 5 years of age, reported rates of cerebral palsy, language impairments, epileptic seizures, and hearing impairments in 8.1%, 7.5%, 7.3%, and 25.8%, respectively [35].

Current literature indicates that WBC in peripheral blood is insufficient for determining alone the need for an LP [36,37]. In line with previous literature, our study found that WBC counts and CRP levels at the onset of BM are not sufficiently helpful, since normal ranges were observed in 48% and 21% of cases, respectively.

GBS emerged as the predominant pathogen. This is not surprising, since GBS is the first cause of BM in our region [38]. Moreover, consistent with previous literature [39], most episodes were due to Gram-positive bacteria, although episodes due to Gram-negative pathogens were substantial and were responsible for the two case fatalities. Indeed, previous studies confirm Gram-negatives (namely *Escherichia coli*) as major causes of BM-related deaths in infants [29,30].

Our findings reveal substantial differences in presenting symptoms of BM between preterm and term infants; this information may guide clinicians in evaluating infants with suspected BM. Krebs and Costa compared clinical signs in 34 neonates with a birth weight under 2500 g and 53 neonates over 2500 g. They found that apnea (20.6%), jaundice (17.6%), and abdominal distension (23.5%) were more prevalent under 2500 g, while irritability (45.3%), seizures (41.5%), and bulging fontanelle (30.2%) were more common over 2500 g [40]. Despite the limitations due to the small sample of neonates in the current study, our study confirms some of these findings, as apnea was frequently seen in preterm infants, while irritability and poor reactivity were predominant in full-term infants.

All infants in our cohort underwent brain ultrasound, and 82% also received MRI; however, brain lesions were detected in only 21% of cases. This finding underscores the need for follow-up in all infants to reveal potential neurodevelopmental impairments. In fact, a systematic literature review indicates that up to 20% of infants with GBS meningitis may develop moderate to severe neurodevelopmental impairments whereas mild impairment may occur in a substantial proportion of children when long-term follow up is performed [41,42].

A regimen of ampicillin, gentamicin, and a third-generation cephalosporin (cefotaxime) is recommended for empirical treatment of EOM, while ampicillin, cefotaxime, and an aminoglycoside are suggested for LOM [18,19,27]. In our study, most empirical treatments involved a semi-synthetic penicillin (ampicillin or oxacillin) combined with an aminoglycoside (gentamicin or amikacin). Glycopeptides, such as vancomycin, were rarely administered in combination with aminoglycosides. Overall, 85% of pathogens were susceptible to the empirical antibiotic regimens used; however, antibiotics effective against Gram-negative pathogens that actually reach therapeutic levels in the CSF were administered in only 82% of episodes.

The strengths of our study include its single-center design and the 13-year period covered, focusing on diagnostic and therapeutic approaches in the first few months of life. Nevertheless, several important limitations should be acknowledged. Firstly, the relatively small sample size and retrospective design restricted our ability to perform detailed analyses, such as correlating specific pathogens with clinical outcomes. Secondly, LP was conducted after empirical antibiotic therapy in 32% of cases, potentially underestimating the real incidence of BM. However, our finding is consistent with an American study showing that 30% of children underwent LP after antibiotic therapy [41]. Lastly, potential misclassification of coagulase-negative staphylococci (CoNS) meningitis due to contamination remains a concern. In our study, there is also a lack of data regarding the long-term neurodevelopmental outcomes of infants, which limits our ability to fully assess the impact of BM in our cohort.

## 5. Conclusions

Bacterial meningitis in neonates and infants in the first three months of life is often underdiagnosed due to the non-specific symptoms, which may delay performing an LP. Moreover, empirical antibiotic therapy is frequently administered before LP, potentially leading to false-negative results. However, molecular tests based on P.C.R. provide a valuable option for diagnosing meningitis in neonates who have already received antibiotics. The empirical therapy recommended by guidelines for suspected meningitis is often omitted or delayed due to the vague clinical presentation. Although our study has a limited sample size, the data presented could be useful for future research aimed at evaluating the feasibility of integrating clinical data with hematological tests to identify patients at higher or lower risk for meningitis. Additionally, future challenges will include implementing therapeutic strategies to reduce long-term sequelae.

## Figures and Tables

**Table 1:** Birth weight, gestational age, blood, CSF culture, relapse, and outcome of the 44 episodes of meningitis.

Episodes (*n* = 44)	BW (Gr)	GA (Weeks)	LOM or EOM	Relapse	BC	CSF Culture	CSF P.C.R.	Duration of Therapy (days)	Brain Lesions or Death
1	740	23	LOM	-	Enterobacter cloacae + Klebsiella pneumoniae	*Enterobacter cloacae*	-	13	Brain lesions * †
2	670	24	LOM	-	Staphylococcus warneri	*Staphylococcus epidermidis MET R*	-	7	-
3	703	24	LOM	-	Staphylococcus aureus	*Staphylococcus aureus*	-	9	Brain lesions *
4	800	24	LOM	-	Acinetobacter baumanni	*Acinetobacter baumanni*	-		-
5	800	24	LOM	Yes	-	*Enterococcus faecalis + Klebsiella pneumoniae*	-	15	Brain lesions * §
6	635	24	LOM	-	Sterile	*-*	Escherichia coli	7	-
7	790	25	LOM	-	Sterile	*Klebsiella pneumoniae*	-	22	-
8	820	25	LOM	-	Klebsiella pneumoniae	*-*	Klebsiella pneumoniae		-
9	880	26	LOM	-	Serratia marcescens	*Serratia marcescens*	-	2	Death
10	450	26	LOM	-	Staphylococcus epidermidis	*Staphylococcus capitis*	-	3	-
11	960	26	LOM	-	-	*-*	Escherichia coli	30	Brain lesions *
12	990	26	LOM	-	Sterile	*Staphylococcus aureus*	-	21	-
13	890	27	LOM	-	Escherichia coli	*Escherichia coli*	-	23	-
14	760	27	LOM	-	Enterobacter cloacae	*Staphylococcus epidermidis MET R*	-	10	Brain lesions †
15	1094	27	LOM	-	Staphylococcus capitis	*Staphylococcus capitis MET R*	-	10	-
16	1223	27	LOM	-	Sterile	*-*	Streptococcus agalactiae	14	Brain lesions * §
17	947	27	LOM	-	Streptococcus agalactiae	*Streptococcus agalactiae*	-	15	-
18	1189	28	LOM	-	Sterile	*Escherichia coli*	-	1	Death
19	934	28	LOM	-	Staphylococcus epidermidis	*Staphylococcus epidermidis*	-	10	-
20	986	28	LOM	-	Sterile	*Enterococcus faecalis + Enterobacter cloacae*	-	25	-
21	986	28	LOM	Yes	-	*Staphylococcus epidermidis MET R*	-		-
22	986	28	LOM	Yes	-	*Enterococcus faecalis*	-		-
23	1495	29	LOM	-	Staphylococcus aureus	*Staphylococcus aureus*	-	14	-
24	1420	29	LOM	-	Enterococcus faecalis + Serratia marcescens	*Serratia marcescens*	-	32	Brain lesions * §
25	1257	29	LOM	-	Staphylococcus epidermidis	*Escherichia coli + Pseudomonas aeruginosa*	-	14	Brain lesions †
26	1840	29	LOM	-	Streptococcus agalactiae	*-*	Streptococcus agalactiae	14	-
27	1808	30	LOM	-	Sterile	*Staphylococcus epidermidis*	-	23	-
28	1192	34	LOM	-	Enterococcus faecalis	*Enterococcus faecalis*	-	16	-
29	2420	34	LOM	-	Streptococcus agalactiae	*Streptococcus agalactiae*	-	11	-
30	2207	35	LOM	-	Klebsiella oxytoca ESBL	*Klebsiella oxytoca ESBL*	-	18	-
31	2895	36	EOM	-	Listeria monocytogenes	*Listeria monocytogenes*	-	15	-
32	2480	37	EOM	-	Streptococcus agalactiae	*-*	Streptococcus agalactiae	14	-
33	3250	38	EOM	-	Streptococcus agalactiae	*Streptococcus agalactiae*	-	17	-
34	2850	38	LOM	-	Sterile	*Escherichia coli*	-	10	-
35	2880	38	LOM	-	Streptococcus pneumoniae	*-*	Streptococcus pneumoniae	20	-
36	3440	39	EOM	-	Listeria monocytogenes	*Listeria monocytogenes*	-	14	-
37	3780	39	EOM	-	Sterile	*Staphylococcus epidermidis*	-		-
38	3760	39	LOM	-	Listeria monocytogenes	*Listeria monocytogenes*	-	21	-
39	4200	39	LOM	-	Sterile	*Corynebacterium tuberculostearicum*	-	9	-
40	2655	39	LOM	-	Staphylococcus epidermidis MET R	*Staphylococcus epidermidis MET R*	-		Brain lesions *
41	2655	39	LOM	Yes	Sterile	*Pseudomonas aeruginosa*	-	24	-
42	3495	40	EOM	-	Staphylococcus hominis	*Pseudomonas aeruginosa*	-	3	-
43	3840	40	EOM	-	Sterile	*Staphylococcus haemolyticus*	-	5	-
44	2790	41	LOM	-	Streptococcus agalactiae	*Streptococcus agalactiae*	-	35	-

EOM: early-onset meningitis; LOM: late-onset meningitis; BW: birth weight; GA: gestational age; BC: blood culture; CSF: cerebrospinal fluid culture; P.C.R.: polymerase chain reaction; MET R: methicillin resistant; IVH: intraventricular hemorrhage. Brain lesions: * ventricular enlargement, † white matter damage, § IVH ≥ 2, hydrocephalus.

**Table 2:** Blood and CSF parameters in preterm and full-term neonates. Data are reported as median and IQR.

		Preterm Infants	Full-Term Infants	*p*-Value
BLOOD	CRP (mg/dL)	4.4 (1.8–9.9)	3.3 (1.3–5.5)	0.22
	WBC (nr/mmc)	10185 (4450–14,345)	6770 (3515–14,922)	0.63
CSF	WBC (nr/uL)	13 (5.5–306)	273.5 (19–1040)	0.13
	Proteins (mg/dL)	224.5 (109–300)	126 (101–230)	0.17
	Glucose (mg/dL)	40 (21.5–70)	37 (20–39)	0.34

CRP: C-reactive protein; WBC: white blood cell count (WBC); CSF: cerebrospinal fluid.

## Data Availability

The raw data supporting the conclusion of this article will be made available by the authors on request.
