# Correction: Buttera et al. Bacterial Meningitis in Infants Under 90 Days of Age: A Retrospective Single-Center Study. *Children* 2024, *11*, 1411

**DOI:** 10.3390/children12111495

**Published:** 2025-11-04

**Authors:** Martina Buttera, Sofia Mazzotti, Tommaso Zini, Lucia Corso, Valeria Dallai, Francesca Miselli, Luca Bedetti, Katia Rossi, Eugenio Spaggiari, Lorenzo Iughetti, Licia Lugli, Alberto Berardi

**Affiliations:** 1 School of Pediatrics Residency, University of Modena and Reggio Emilia, 41224 Modena, Italy; 297187@studenti.unimore.it (M.B.); lorenzo.iughetti@unimore.it (L.I.); 2 Pediatric Unit, Arcispedale Santa Maria Nuova, University of Modena and Reggio Emilia, 41224 Modena, Italy; tommaso.zini@unimore.it; 3 Degree Program in Medicine and Surgery, University of Modena and Reggio Emilia, 41224 Modena, Italy; 213636@studenti.unimore.it; 4 Neonatal Intensive Care Unit, University Hospital of Modena, 41224 Modena, Italy; 79638@studenti.unimore.it (F.M.); bedetti.luca@aou.mo.it (L.B.); rossi.katia@aou.mo.it (K.R.); spaggiari.eugenio@aou.mo.it (E.S.); lugli.licia@aou.mo.it (L.L.); alberto.berardi@unimore.it (A.B.); 5 Pediatric Unit, University Hospital of Modena, 41124 Modena, Italy

In the original publication [1], there was a mistake in Figure 1 as published. An error was made in counting brain lesions in full-term infants with LOM (n = 1, not n = 8). The corrected Figure 1 appears below. The authors state that the scientific conclusions are unaffected. This correction was approved by the Academic Editor. The original publication has also been updated.

## Figures and Tables

**Figure 1:**
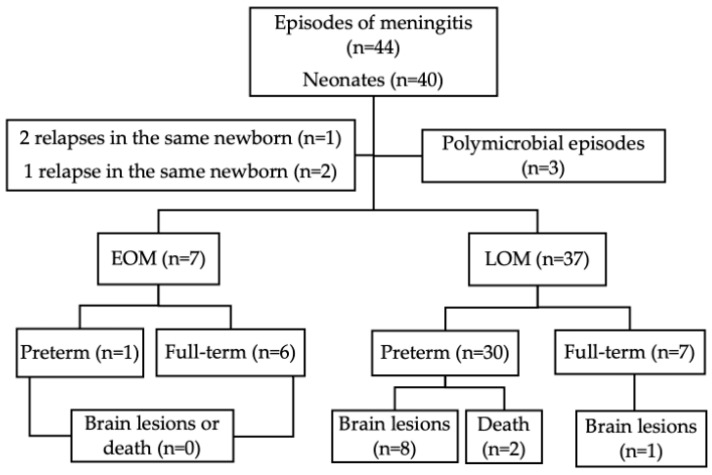
Main characteristics of the study population. EOM: early-onset meningitis; LOM: late-onset meningitis.
